# Understanding the Technological Landscape of Home Health Aides: Scoping Literature Review and a Landscape Analysis of Existing mHealth Apps

**DOI:** 10.2196/39997

**Published:** 2022-11-11

**Authors:** Elizabeth Fong-Chy Kuo, Jacklyn Cho, Iredia Olaye, Diana Delgado, Nicola Dell, Madeline R Sterling

**Affiliations:** 1 Department of Information Science Jacobs Technion-Cornell Institute Cornell Tech New York, NY United States; 2 Division of General Internal Medicine Department of Medicine Weill Cornell Medicine New York, NY United States

**Keywords:** home health aides, home care services, mobile health, mHealth, mobile apps, mobile phone apps, smartphones, educational technology, technology, mobile phone

## Abstract

**Background:**

Home health aides (HHAs) provide necessary hands-on care to older adults and those with chronic conditions in their homes. Despite their integral role, HHAs experience numerous challenges in their work, including their ability to communicate with other health care professionals about patient care while caring for patients and access to educational resources. Although technological interventions have the potential to address these challenges, little is known about the technological landscape and existing technology-based interventions designed for and used by this workforce.

**Objective:**

We conducted a scoping review of the scientific literature to identify existing studies that have described, designed, deployed, or tested technology-based tools and apps intended for use by HHAs to care for patients at home. To complement our literature review, we conducted a landscape analysis of existing mobile apps intended for HHAs providing in-home care.

**Methods:**

We searched the following databases from their inception to October 2020: Ovid MEDLINE, Ovid Embase, Cochrane Library, and CINAHL (EBSCO). A total of 3 researchers screened the yield using prespecified inclusion and exclusion criteria. In addition, 4 researchers independently reviewed these articles, and a fifth researcher arbitrated when needed. Among studies that met the inclusion criteria, data were extracted and summarized narratively. An analysis of mobile health apps designed for HHAs was performed using a predefined set of terms to search Google Play and Apple App stores. Overall, 2 researchers independently screened the resulting apps, and those that met the inclusion criteria were categorized according to their intended purpose and functionality.

**Results:**

Of the 8643 studies retrieved, 182 (2.11%) underwent full-text review, and 4.9% (9/182) met our inclusion criteria. Approximately half (4/9, 44%) of the studies were descriptive in nature, proposing technology-based systems (eg, web portals and dashboards) or prototypes without a technical or user-based evaluation of the technology. In most (7/9, 78%) papers, HHAs were just one of several users and not the sole or primary intended users of the technology. Our review of mobile apps yielded 166 Android and iOS apps, of which 48 (29%) met the inclusion criteria. These apps provided HHAs with one or more of the following functions: electronic visit verification (29/48, 60%), clocking in and out (23/48, 48%), documentation (22/48, 46%), task checklist (19/48, 40%), communication between HHA and agency (14/48, 29%), patient information (6/48, 13%), resources (5/48, 10%), and communication between HHA and patients (4/48, 8%). Of the 48 apps, 25 (52%) performed monitoring functions, 4 (8%) performed supporting functions, and 19 (40%) performed both.

**Conclusions:**

A limited number of studies and mobile apps have been designed to support HHAs in their work. Further research and rigorous evaluation of technology-based tools are needed to assess their impact on the work HHAs provide in patient’s homes.

## Introduction

### Background: Home Health Aides

By 2060, the number of Americans aged >65 years is projected to reach approximately 95 million, making up almost one-fourth of the population in the United States. Most older adults, including those with multiple chronic conditions, prefer to stay in their homes and communities for as long as they can and avoid nursing homes, a concept referred to as “aging in place.” To do so, they require help at home from family caregivers and home health aides (HHAs). HHAs represent the sixth fastest growing occupation in the United States; at present, there are 2.3 million HHAs in the United States and are expected to grow by 1.5 million by 2030 [1]. Largely employed by home care agencies, HHAs are trained and certified health professionals who provide assistance with personal care (such as activities of daily living [eg, bathing and dressing] and instrumental activities of daily living [eg, preparing meals, cleaning, and shopping], emotional support, and medically oriented care [eg, taking vital signs and medication reminders]) to older adults and those with chronic conditions at home [2]. Unlike other health professionals such as physicians, nurses, and physical therapists, HHAs interact with patients on a daily or near-daily basis, which gives them a unique vantage point from which to observe, support, and advise patients [3]. Therefore, they are often referred to as the “eyes and ears” for patients and the medical team in the home.

### Professional Challenges on the Job

However, despite being integral to patient care, HHAs are an overlooked and underutilized group of health care professionals who experience challenges in caring for patients at home. Most women and minorities of color who earn low wages work long hours, have erratic schedules, and have limited opportunities for career advancement [4]. Studies have found that HHAs increasingly care for medically complex patients with a high burden of chronic diseases [5,6]. Despite their observations and insights into patients’ health, they are rarely considered a part of the patient’s medical team. In addition, when they try to report information, convey their concerns, have questions, or need advice, they have difficulty reaching their supervisors via phone. Furthermore, formal technology systems are lacking in conveying the data they collect to other health care professionals. eHealth, which refers to health services that are delivered through the internet or other technologies [7], mobile health (mHealth), and telehealth, which are both subsets of eHealth, have the potential to fill this gap and benefit HHAs. Technology can connect multiple aspects of the health care system (physicians, patients, staff, and HHAs) and increase the efficiency of health care delivery. Although eHealth interventions can be challenging to implement because of their complexity, the integration of technology may ultimately make HHA jobs easier [8].

Finally, although HHAs receive training for certification and maintenance, many of their courses are general and not disease specific, which may not meet the clinical needs of the older adults [9]. Qualitative studies have shown that the workforce wants and benefits from technological systems to address these issues. For example, when HHAs monitor their patients’ blood pressure, a technological system that allows for the measurement, recording, and transmission of these data to other providers would be useful, especially when the values are abnormal. Similarly, when caring for patients and questions arise about their conditions, many HHAs rely on past clinical experiences or “Google” their questions using their personal devices. Instead, HHAs might prefer to have access to disease-specific (eg, stroke) information on their personal devices that they can reference while at work.

### Objective

To meet these needs and inform future technological innovations for this workforce, a better understanding of the technology landscape is needed [10]. Herein, we conducted the first scoping review of the scientific literature to identify existing studies that have described, designed, deployed, or tested technology-based tools and apps that are intended for use by HHAs as they care for older adults or those with chronic conditions. To complement our literature review and provide additional context, we also conducted a landscape analysis of existing mobile apps pertaining to HHAs and the care they provide in the home environment.

### Methods: Scoping Review

This scoping review is reported in line with the PRISMA-ScR (Preferred Reporting Items for Systematic Reviews and Meta-Analyses extension for Scoping Reviews) guidance [11].

#### Guiding Framework

We conducted a scoping review using the 5-stage framework developed by Arksey and O’Malley [12]. The 5 stages include (1) identification of the research question, (2) identification of relevant studies, (3) selection of relevant studies for the review, (4) charting information and data from the selected literature, and (5) summarizing and reporting the results of the review.

#### Search Strategy

A medical librarian (DD) performed a comprehensive literature search on October 28, 2020, of Ovid MEDLINE(R) ALL, from 1946 to October 27, 2020, Ovid Embase (from 1974 to October 27, 2020), Cochrane Library (Cochrane Database of Systematic Reviews, Cochrane Central Register of Controlled Trials, and Cochrane Methodology Register), and CINAHL (EBSCO) from inception to October 2020. The first search was conducted using Ovid MEDLINE. Subject headings and keywords were adapted for other databases. No restrictions were applied on language, publication date, or article type. Additional records were identified by reviewing reference lists and using the “Cited by” and “View references” features in Scopus of the included studies. The full set of search terms for Ovid MEDLINE is presented in Multimedia Appendix 1.

#### Inclusion and Exclusion Criteria

This review was limited to studies that focused on technology-based tools, innovations, or interventions intended to be used by home health care workers (including HHAs, attendants, and personal care aides). Studies can be descriptive in nature (eg, overview of technology design), quasi-experimental, or randomized controlled trials. Only peer-reviewed studies published in the English language were included. Qualitative studies that did not discuss or propose an intervention, reviews, editorials, or scientific meeting abstracts were excluded. Studies that focused on other people who provide care at home (eg, nurses or family caregivers) were excluded. Studies that were conducted in nursing homes, long-term care centers, and acute rehabilitation centers were also excluded.

#### Selection of Studies

All studies identified following the database search were uploaded to the web-based systematic review software package Covidence (Veritas Health Innovation). First, the title and abstract reviews of all studies were completed independently by 3 authors (JC, IO, and ND). Disagreements were discussed and resolved through consensus. A record was kept of all the studies excluded and the reason for exclusion in Covidence. All studies that met the inclusion criteria (189 studies) went through a full-text screening process by the 4 authors independently (JC, IO, EFK, and ND), and any disagreements on the eligibility of the studies were reviewed by a fifth author (MRS).

#### Data Extraction

Data from the included studies were extracted using the following categories: (1) author, (2) country, (3) year of publication, (4) title of the study, (5) journal, (6) contribution, (7) technology innovation, (8) intended users, (9) study objective and systems goals, and (10) evaluation and assessment of innovation.

## Results

### Study Characteristics

**Figure 1:**
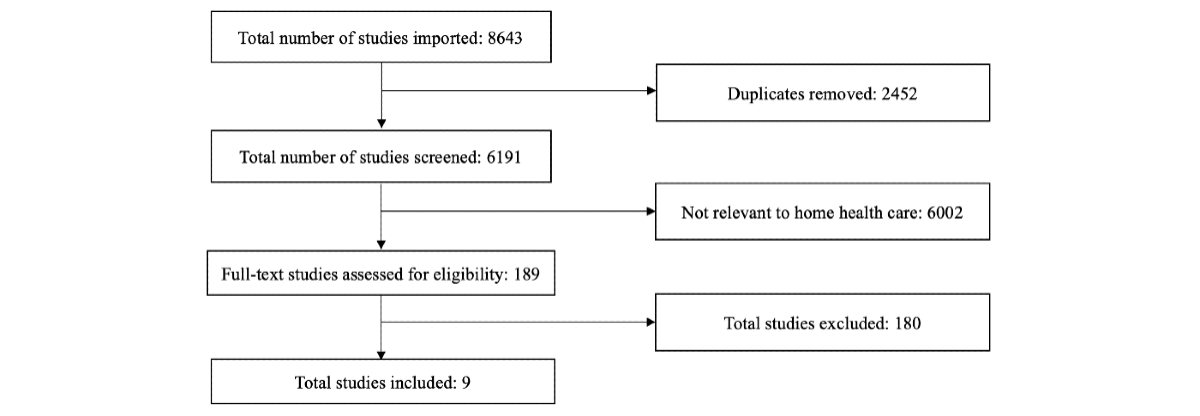
PRISMA (Preferred Reporting Items for Systematic Reviews and Meta-Analyses) flow diagram for the scoping literature review.

In total, 8643 studies were imported from our search of the peer-reviewed literature. Among these 8643 studies, 2452 (28.36%) were excluded because they were duplicates. We screened 6191 abstracts and excluded 6002 (96.94%) studies because they were not relevant to home health care. A total of 189 full-text studies were assessed for eligibility, and 7 studies were included. A medical librarian (DD) identified 13 additional studies from the citation chasing process; among these, 2 studies met the inclusion criteria. Taken together, we identified 9 full-text studies that met the inclusion criteria (Figure 1).

The characteristics of the 9 included studies are presented in Table 1 (Multimedia Appendix 2 [13-21]). The studies were published between 2004 and 2018 in journals that focused on technology, computer science, home and long-term care, and gerontology or aging. Most studies were conducted in Europe (5/9, 56%), whereas one-third (3/9, 33%) were conducted in the United States and 11% (1/9) in Japan. More than half (6/9, 67%) of the studies were descriptive in nature, proposing technology-based systems (eg, web portal or dashboard) or prototypes without a technical or user-based evaluation. Of the 3 studies that included evaluations, all but one (1/3, 33%; Danilovich et al [21]) were design prototypes or feasibility pilot studies with limited data collection on the users (eg, nurses, family caregivers, or HHAs) of the technology or on patients. Of the 9 included studies, most (n=7, 78%) evaluated HHAs as just one of several caregivers or health professionals as intended end users, rather than as primary users. Most (6/9, 67%) of the technological interventions were web based, 22% (2/9) of studies described mobile apps, and 11% (1/9) of studies tested a DVD. We discuss these studies in detail in the following sections.

**Table 1:** Results from the scoping review of literature.

Author, year, and country^a^	Study title	Technology innovation	Intended users	HHA^b^ role
Ogawa et al [13], 2004; Japan	A Java mobile phone-based “home helper” care report creation support system	Java mobile phone–based care report creation support system	Home helpers	Primary
Scandurra et al [14], 2006; Sweden	Visualisation and interaction design solutions to address specific demands in shared home care	Design prototype based on participatory design	GP^c^, DN^d^, and HHS^e^ personnel	Peripheral; one of many
Paganelli et al [15], 2011; Italy	An ontology-based system for context-aware and configurable services to support home-based continuous care	Emilia Romagna Mobile Health Assistance Network (ERMHAN) service platform	Patients, family members, home care teams (clinicians, GPs, nurses, etc), and social community members (eg, social workers and volunteers)	Peripheral; one of many
Page et al [16], 2012; the United States	Improving care delivery using health information technology in the home care setting: development of the home continuation care dashboard	Web dashboard intended to bridge the gap between physicians and home care case managers	Physicians, home care case managers, patients, and caregivers	Peripheral; one of many
De Backere et al [17], 2016; Belgium	The OCareCloudS project: toward organizing care through trusted cloud services	OCarePlatform and cloud-based semantic system to offer information and knowledge-based services for older people and their informal and formal caregivers	Older patients residing at home and informal and formal caregivers	Secondary; 1 of 3 (patient is primary)
De Backere et al [18], 2017; Belgium	The OCarePlatform: a context-aware system to support independent living	Sensor-based in-home system	Multiple formal and informal caregivers involved in a patient’s care	Peripheral; one of many
Danilovich et al [19], 2017; the United States	Design and development of a mobile exercise application for home care aides and older adult Medicaid home and community-based clients	Mobile exercise app. Content of the program itself seems static. Minimal data entry about patient (pain and mood)	Home HCA^f^ and patients	Secondary; 1 of 2 (patient is primary)
Bourikas et al [20], 2017; the United Kingdom	Elderly support to inspired ageing (ESTIA)	Elderly Support to Inspired Ageing platform that enables medical and background information to be combined into a single server	Family, volunteers, older people, home care aides, hospitals	Primary
Danilovich et al [21], 2017; the United States	Translating Strong for Life into the Community Care Program: Lessons Learned	SFL^g^: Resistance Exercise Intervention: 35-minute DVD on warm-up and upper and lower extremity exercises for homebound older adult clients	Home HCA and patients	Secondary; 1 of 2 (patient is primary)

^a^Studies are listed in chronological order based on the year published.

^b^HHA: home health aide.

^c^GP: general practitioner.

^d^DN: district nurse.

^e^HHS: home help service.

^f^HCA: health care aides.

^g^SFL: strong for life.

### Intended Users of Technology

HHAs were the intended users of the technology for 33% (3/9) proposed technology interventions. Of these 3 studies, only 1 (33%) study by Ogawa et al [13] exclusively focused on supporting HHAs in their work, whereas the remaining 2 (67%) studies included HHAs as peripheral users of the technology with the larger goal of supporting the patients. In a study of 21 home helpers (eg, HHAs) in Japan, Ogawa et al [13] described a Java mobile phone–based system intended for HHAs to use when caring for older adults at home. The system aimed to reduce the amount of time and technical challenges for HHAs reporting to their agencies and patients. In contrast, 2 studies by Danilovich et al [19,21] designed technology for HHAs to use; HHAs were one of several stakeholders involved in intervention development and deployment, and the primary focus of the technology was ultimately the patient. For example, in one of their 2017 studies, Danilovich et al [19] designed, developed, and piloted a mobile exercise app (app and videos) for frail older patients, and HHAs and physical therapists were trained on how to help these patients use the app. In a separate study, Danilovich et al [21] trained home care aides in an evidence-based resistance exercise program that consisted of workout DVDs. However, as in the previous study, the intended users of the exercise DVD program were homebound and community-dwelling patients.

The remaining 67% (6/9) of studies examined technology tools that were not designed primarily for HHAs. HHAs were one of several types of users. Although these tools supported HHAs, they targeted patients and general members of the health care team, such as physicians, nurses, and physical therapists, as the main users. For example, Page et al [16] described the development of a web-portal home continuation care dashboard to facilitate communication among case managers, physicians, patients, and their caregivers at home.

### Purpose of Technology

All (9/9, 100%) studies aimed to support HHAs in caring for patients at home. There was a wide range of distinct purposes of the technology proposed and tested. Overall, 78% (7/9) of studies proposed and described digital software platforms, ranging from web-based platforms to enhance communication in the home environment to a sensor-based in-home tool to alert caregivers of their patients’ falls and physical injuries. Only the study by Ogawa et al [13] focused on designing and pilot-testing a Java mobile phone–based system to facilitate data entry and documentation exclusively for HHAs. The remaining platforms were designed to facilitate communication and coordination among patients and members of their health care team. These members included physicians, nurses, case managers, HHAs, and the patient’s family members.

A total of 22% (2/9) of studies designed and tested a technological intervention intended to improve patients’ physical mobility. Both studies by Danilovich et al [19,21] used technology (mobile apps and DVDs) to train HHAs on exercise regimens, which could benefit patients (mobility) and HHAs (job satisfaction). Specifically, Danilovich et al [19] developed and pilot-tested a mobile exercise app with HHA-patient dyads. The mobile app presented users (eg, older patients and their HHAs) with several exercise videos filmed by the researchers and HHAs. These videos are downloaded onto the app so that users can access the app without the internet. On completing the exercises, users are prompted to update their progress on the app. Although the researchers included HHAs as a key group of stakeholders who could assist patients with the app, the app was intended for patients and did not provide direct assistance to support HHAs’ work.

### Study Design and User Evaluation

Of the 9 studies, 6 (67%) presented descriptions of the proposed technology and system. None of these studies included evaluations of the technology or data on feedback from the intended users, HHAs. A study discussed the development and testing of a system prototype among home help service personnel, nurses, and general practitioners. However, no follow-up user evaluations or deployment data were evaluated.

Only 22% (2/9) of studies collected and reported quantitative and qualitative data on user evaluation and deployment efforts from the perspective of users, one of whom was HHAs. These evaluation efforts focused on overall program satisfaction. For example, a study by Danilovich et al [21] conducted a mixed methods randomized controlled trial and specifically focused on examining the effect of a technology-based exercise program on home care aides’ perceptions of job satisfaction, achievement, and recognition. In this study, the researchers assigned 17 and 15 HHAs to the intervention and control groups, respectively. The intervention group received resistance bands and exercise programs in a DVD-based format for older adults to complete on days when the HHAs did not visit. The researchers compared preintervention and postintervention scores to assess the effectiveness of the resistance exercise intervention. Quantitative and qualitative assessments were conducted and evaluated using validated instruments (ie, Job in General and Work on Present Job scales) and blinded field observations by the researchers.

In another study by Danilovich et al [19], the authors developed and pilot-tested a novel mobile exercise app to engage older adults in physical activity. A total of 5 HHA-patient dyads were recruited to participate in the study. The participants provided quantitative and qualitative feedback via written questionnaires and semistructured interviews. The outcomes assessed were usability evaluations focusing on 3 domains: system usefulness, information quality, and interface quality. The participants provided further feedback on the functionality and aesthetics of the mobile app.

### Methods: Landscape Analysis

On the basis of paucity of results from the scoping review, we conducted a landscape analysis of existing mobile apps that were designed for HHAs and could potentially assist them in their work caring for patients in the home.

#### Search Strategy

Two authors (EFK and JC) searched for existing mHealth apps created for HHAs on the Google Play store (for Android apps) and the Apple App store (for iOS apps) using a predefined set of terms (Multimedia Appendix 3).

#### Screening and Data Extraction

Our inclusion criteria for mobile apps included apps that (1) were available on the iOS Apple or Google Play stores, (2) were primarily designed for HHAs, and (3) supported HHAs with their work in patients’ homes. For example, we included apps with features for documentation, communication, and training. These are resources that HHAs may use while working directly with patients.

Our initial search yielded 686 Android apps and 289 Apple apps that were screened for inclusion. We created a custom-built Python script to automatically save all the search results as a list, which facilitated further analysis. In our first pass, we removed apps that were clearly not relevant to HHAs (eg, patient self-tracking apps, radio stations, or self-help books) This yielded 175 apps: 148 Android and 27 Apple apps. For each app, we collected the app name, ID, year released, last year updated, number of downloads, app description, and number of reviews. We then removed an additional 8 apps that were duplicated in the data set (ie, had both Android and iOS versions). A total of 167 apps underwent independent review by 2 authors (EFK and JC), who examined each app’s descriptions (found on the Google Play or the iOS Apple App stores) for their intended users (eg, HHA, medical professionals including HHA, or nurses) and purposes (eg, connecting providers with patients, providing task checklists, and GPS tracking). Apps that did not make HHAs one of the primary users were excluded.

After doing so, a total of 67 mobile apps met our inclusion criteria for further review.

We verified the completeness of the resulting set of apps in 2 ways. (1) For each relevant app, we used search engine optimization software (Semrush) that given the name and URL of a relevant app, provided a list of “competitor” apps that would be likely serve the same purpose or provide similar functionality. We reviewed the suggested competitor apps for all HHA-relevant apps to confirm whether they were already present in the set of apps or assess whether they met the inclusion criteria. (2) In addition, we used the built-in “recommended app” features provided by both Google Play and Apple App stores. We entered the name of each HHA-relevant app and noted any alternative or similar apps that were recommended by each platform. We then checked these recommended apps to see if they were already in our data set or assessed whether they should be included. Neither of these processes yielded new apps that were not already present in our data set, which increased the confidence that our search process discovered all relevant apps.

## Results

### Characteristics of the Included Mobile Apps

**Figure 2:**
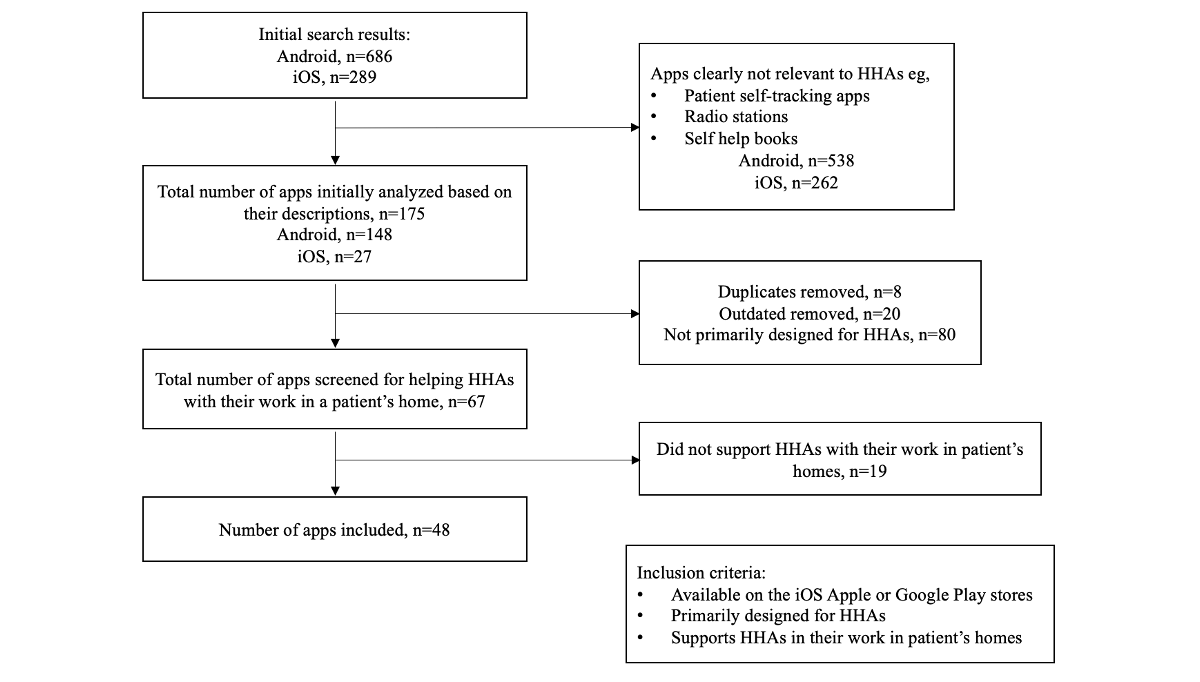
Flow diagram for the landscape analysis of mobile health apps. HHA: home health aide.

Since we sought to study apps that assisted HHAs with their work in a patient’s home, apps that only served HHAs before or after their patient visit were eliminated. Through our previous categorizations based on each mobile app’s description, we included apps that performed at least one of the following functions: (1) allowed HHAs to access their task checklist; (2) allowed HHAs to document their work of the day; (3) provided a place for HHAs to access their patient’s information; (4) facilitated communication between an HHA and their agency; (5) facilitated communication between an HHA and their patient; (6) provided resources such as training courses, information, and so on for HHAs; (7) helped with electronic visit verification; and (8) assisted HHAs with clocking in and out. After applying these criteria to the 67 apps, 48 (72%) remained that performed one or more of the 8 core functions (Figure 2).

An overview of the characteristics of these 48 unique apps is presented in Table 2 (Multimedia Appendix 4). The majority of apps studied assisted with electronic visit verification (29/48, 60%); fewer apps provided means for communication between HHAs and patients (4/48, 8%) and resources for HHAs (5/48, 10%). Other notable functions that apps had were clocking in and out of shifts (23/48, 48%), documenting work performed by the HHA (22/48, 46%), allowing access to a task checklist for the day (19/48, 40%), facilitating communication between the HHA and the agency (14/48, 29%), and providing patient information (6/48, 13%). Each app may have more than one characteristic. We further categorized the 48 apps according to their primary purpose of monitoring HHAs, supporting HHAs, or both. We defined a monitoring app as one that helps an agency or employer keep track of HHAs’ tasks or their location in the patient’s home. We defined a supporting app as one that provides various resources or training tools for the HHA, gives the HHA information on their patient, or assists with communication between the HHA and their agency or patient.

**Table 2:** Results from the landscape analysis of mobile apps.

Name of the mobile app^a^	Year	Type	Primary users	Objective
Domiciliary Care Toolkit	2014	Android	Home care providers (including HCWs^b^)	Supporting
HHAeXchange	2014	Android	HCW	Both
Verify Centre Home Health	2015	Android	HCW	Both
Alora Plus	2016	Android	HCW	Both
Connected Home Care	2016	Android	HCW	Both
Electronic Visit Verification	2016	Android	HCW	Monitoring
FreedomCare Plus	2016	Android	HCW	Monitoring
MedFlyt	2016	Android	Home care providers (including HCWs)	Both
PointClickCare Care at Home	2016	Android	HCW	Monitoring
CareConnect	2017	Android	HCW	Supporting
Axxess HomeCare	2017	Android	HCW	Both
Caretap EVV	2017	Android	Home care providers (including HCWs)	Monitoring
DCI Mobile EVV	2017	Android	HCW	Both
eRSP Mobile Connect	2017	Android	Home care providers (including HCWs)	Both
FormDox EVV for Aides	2017	Android	HCW	Both
Ally Home Care	2018	Android	HCW	Monitoring
August Systems Mobile for Caregivers	2018	Android	HCW	Both
AuthentiCare 2.0	2018	Android	HCW	Monitoring
ClearCareGo Caregiver	2018	Android	HCW	Monitoring
CliniqOS	2018	Android	Home care providers (including HCWs)	Both
CrescendoConnect	2018	Android	Home care providers (not specific to HCWs)	Both
Domiciliary Care Worker Gweithiwr Gofal Cartref	2018	Android	HCW	Supporting
Helpers Home Care	2018	Android	Home care providers (including HCWs)	Both
My EVV	2018	Android	HCW	Monitoring
MyEzcare—EVV	2018	Android	HCW	Monitoring
Mobile Caregiver+	2018	Android	HCW	Monitoring
Honor Care Pro	2018	Android	HCW	Both
UCP Caregiver Staffing	2018	iOS	HCW	Monitoring
BarbaraKares	2019	Android	HCW	Monitoring
CareTime	2019	Android	HCW	Monitoring
Cashe EVV	2019	Android	HCW	Both
KorEvv	2019	Android	HCW	Monitoring
MatrixCare for Home Care	2019	Android	HCW	Both
myHRresults—At Work	2019	Android	HCW	Monitoring
SwyftOps—Caregiver App	2019	Android	HCW	Monitoring
Vertex EVV	2019	Android	HCW	Monitoring
HomecareGPS Mobile	2019	iOS	HCW	Monitoring
ServTracker Mobile Home Care	2019	iOS	HCW	Monitoring
Moravia Shifts	2020	Android	HCW	Monitoring
Netsmart Homecare Mobile Phone	2020	Android	HCW	Both
BAYADA Home	2021	Android	HCW	Monitoring
Careswitch	2021	Android	HCW	Both
Visit Wizard Mobile	—^c^	Android	HCW	Both
Best Care	—	Android	HCW	Monitoring
Caregiver App	—	Android	HCW	Monitoring
Caregiver Cloud Training	—	Android	Home care providers (including HCWs)	Supporting
Time4Care	—	Android	HCW	Monitoring
ViolaCare	—	Android	HCW	Monitoring

^a^Apps are listed in chronological order based on the year created or last updated.

^b^HCW: home care worker.

^c^Missing information.

Of the 48 apps, 25 (52%) apps focused on monitoring functions, 4 (8%) apps provided supporting functions, and 19 (40%) apps provided both. Of the 48 apps, 34 (71%) were developed and sponsored by software companies, 9 (19%) were developed and sponsored by individual home care agencies, 1 (2%) by government-partnered software companies, and 1 (2%) by government-partnered agencies. Specific developer information could not be found for 6% (3/48) of the apps. The apps that were developed between the years 2014 and 2021 were from 5 countries; most apps were from the United States (41/48, 85%), with the remainder from the United Kingdom, Ireland, Canada, and Ethiopia. The number of downloads ranged from 5 to >100,000, and user ratings ranged from 2 out of 5 stars to 5 out of 5 stars.

### Apps That Only Monitor HHAs in the Home

The most common feature provided by the apps was monitoring of HHAs at home (25/48, 52%). This included monitoring whether HHAs arrived at the patient’s home on time by logging HHAs’ work hours (16/25, 64%), keeping track of HHAs’ tasks (11/25, 44%), reporting their real-time GPS location in the patient’s home via electronic visit verification (17/25, 68%), and documenting information about the patient (11/25, 44%).

**Figure 3:**
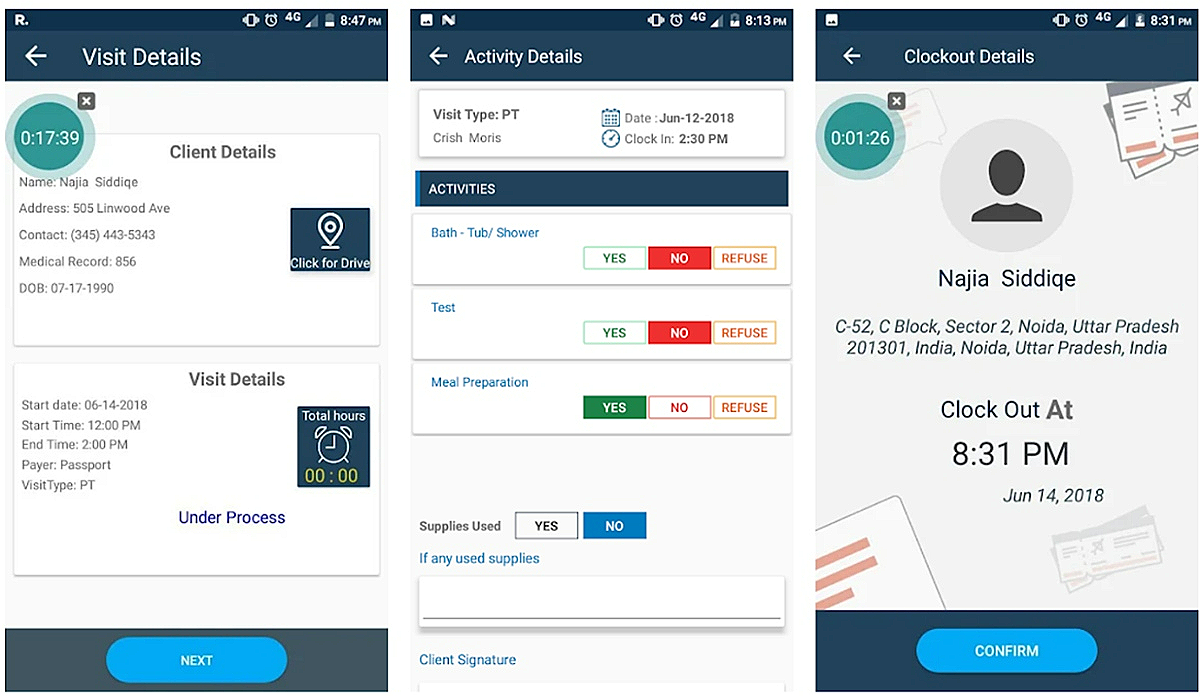
FormDox EVV interface.

For example, FormDox EVV (Figure 3), which was developed by FormDox Technology Solution, includes features such as electronic visit verification and documentation services. The app focuses on assisting agencies by tracking their HHAs and collecting data on HHA performance. It was rated 4.4 out of 5 stars by the users (Multimedia Appendix 4).

**Figure 4:**
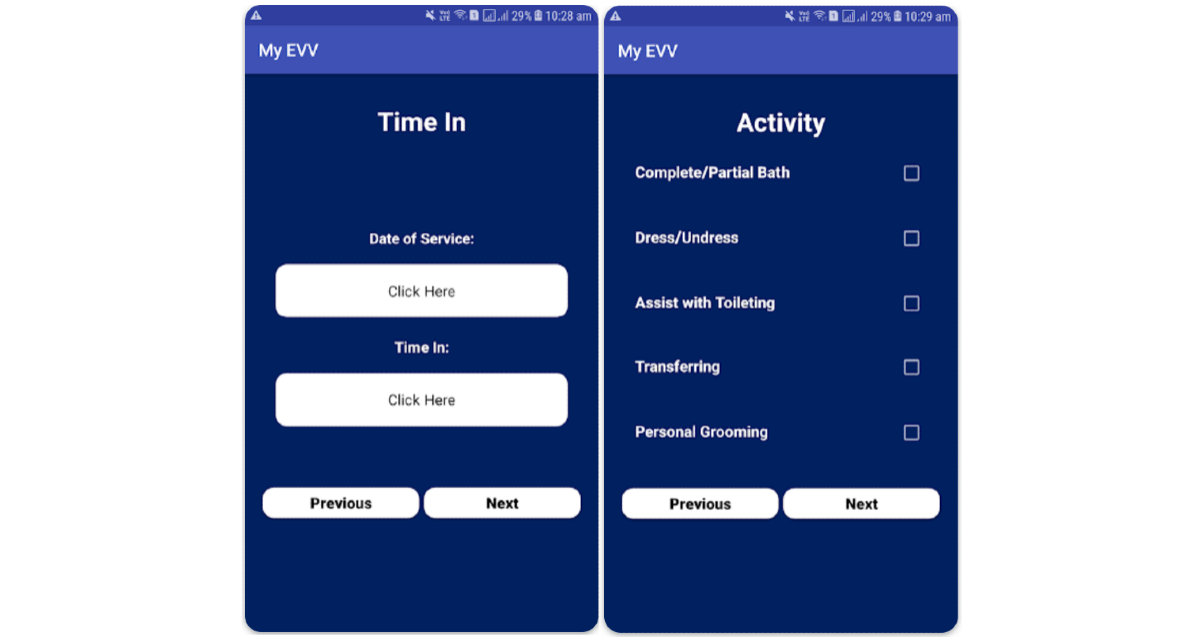
My EVV interface.

Another example is My EVV (Figure 4), which is an app that allows HHAs to clock in and out of shifts electronically and allows HHAs to document services provided to the patient. The app received 3.9 stars on the Google Play store and has been downloaded >10,000 times (Multimedia Appendix 4).

### Apps That Only Support HHAs at Home as They Provide Care

Of the 48 apps, 4 (8%) focused solely on providing support for HHAs. Of the 4 apps, 3 (75%) them provided information or training resources for HHAs and 1 (25%) app assisted with communication between HHAs and their agencies. However, none of the apps included other supporting functions, such as assisting with communication between HHAs and their patients or providing information about the patient to the HHA.

**Figure 5:**
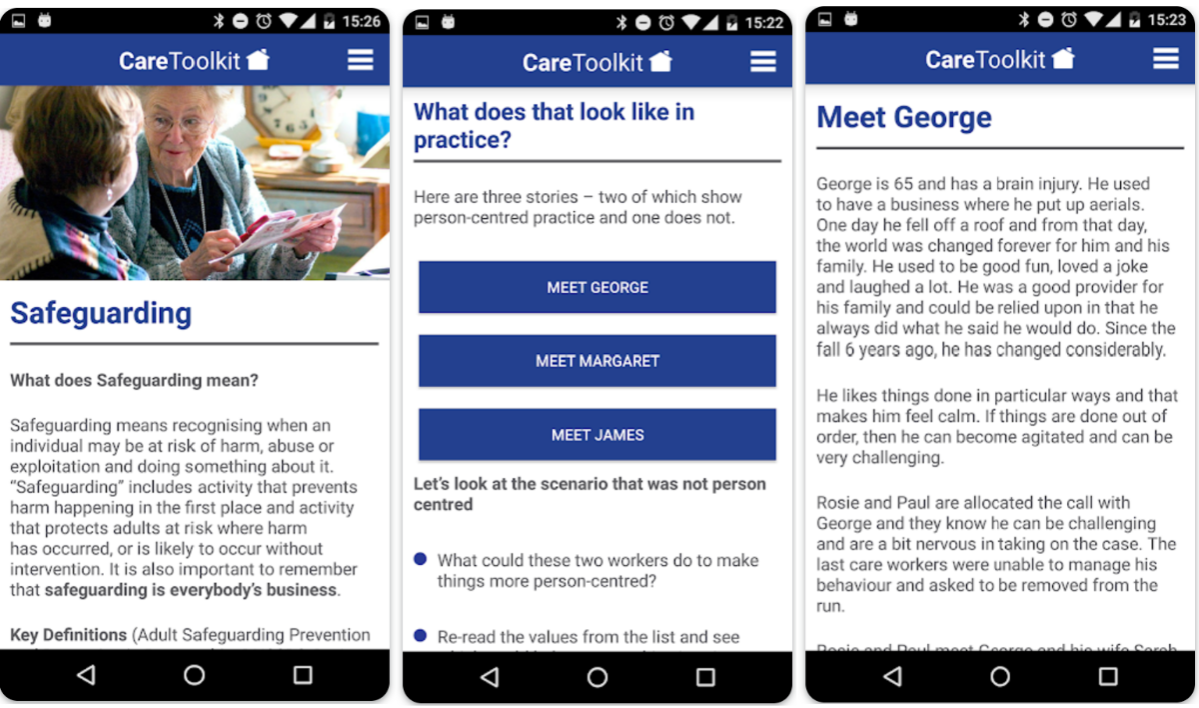
Domiciliary Toolkit interface.

An example of a supporting app is the Domiciliary Care Toolkit (Figure 5). This app was developed by the Northern Ireland Social Care Council in partnership with care providers. It provides guidelines for HHAs on how to conduct clinical tasks as well as resources on how to handle patients with dementia and delirium. The app is unique in that there are no functions that track HHAs, and its only purpose is to serve as a reference tool. It describes the issues an HHA may face and offers possible solutions through patient examples.

### Apps That Both Monitor and Support HHAs

Of the 48 apps, 19 (40%) were apps that both supported and monitored HHAs. The 19 apps included features for keeping track of tasks (n=8, 42%), for HHAs to use for documentation (n=11, 58%), for electronic visit verification (n=12, 63%), for assisting with clocking in and out (n=7, 37%), for HHAs to find information about the patient (n=6, 32%), for facilitating communication between the HHA and patient (n=4, 21%), for facilitating communication between the HHA and agency (n=12, 63%), and for resources (n=1, 5%).

**Figure 6:**
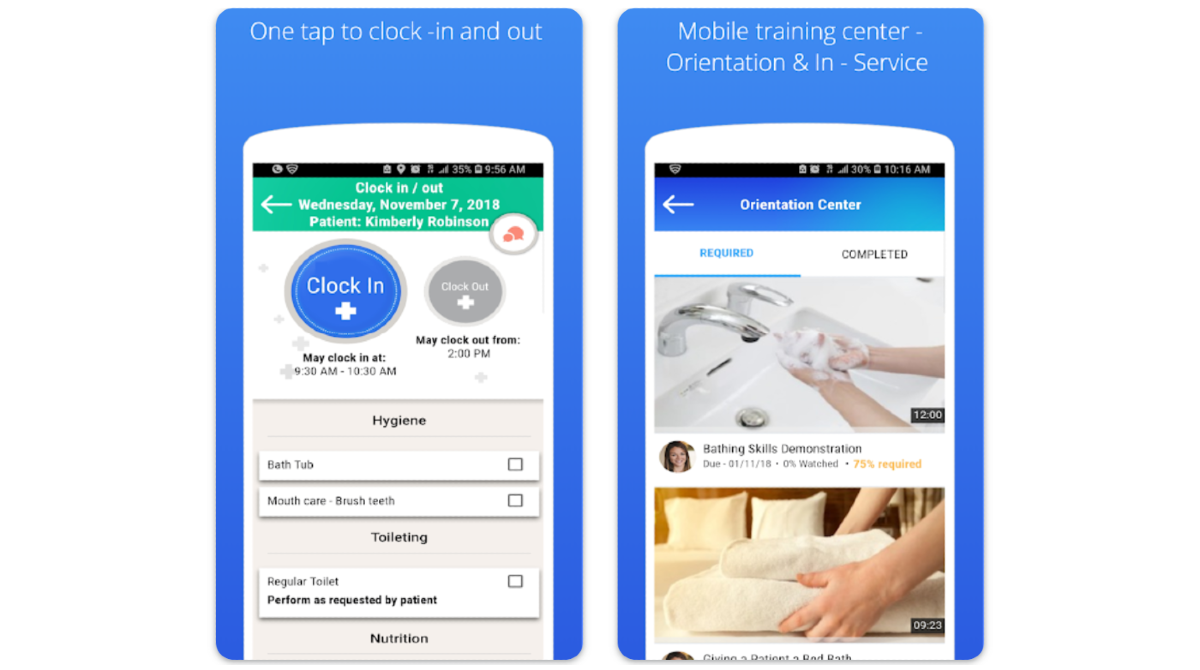
MedFlyt interface.

For example, the app MedFlyt (Figure 6) allows agencies to not only monitor HHAs through documentation features and reminders to clock in and out but also support HHAs with web-based training courses and communication (eg, instant messaging between HHA and the agency). With 2466 reviews and 4.7 stars, the app is one of the most highly rated apps of the 48 we reviewed (Table 2).

## Discussion

### Main Findings

Our findings illustrate the need for increased research on technological interventions for HHAs and the further development of mobile technologies that support HHAs with their work in patients’ homes. The scoping review of the peer-reviewed literature yielded only 9 studies, and in most of them, HHAs were not the primary intended users of the technology. In addition, very few studies have assessed the feasibility and effectiveness of this technology among HHAs. The landscape analysis revealed only 4 existing apps that were solely focused on supporting HHAs, with most apps designed for agencies to monitor HHAs rather than assisting them with their work. The lack of studies from the scoping review that included data on user feedback and the dearth of research on how mobile technologies impact HHAs’ work suggests an urgent need for research that rigorously evaluates technology-based tools to measure their effect on HHAs’ caregiving in patients’ homes.

Research in the field of human-computer interaction has long acknowledged the importance of actively engaging the eventual users of technologies in their design [22], such as via participatory or ethnographic methods that enable designers to deeply understand the context and problems being addressed [23-25]. Without taking the time to learn people’s current practices and understand their perspectives, priorities, and values, designers run the risk of building technologies that are not appropriate or usable and fail to meet people’s true needs. The stakes are particularly high in in-home health care, where poorly designed technologies might have a negative impact not only on HHAs but also on patient care.

Although prior work [26] suggested that HHAs are eager to play an active role in the design of technologies, none of the 9 papers in our scoping review discussed engaging with HHAs to deeply understand their needs and workflows before building an intervention. Moreover, none of the studies presented a long-term evaluation of the impact of the intervention on the HHAs’ workflow or patient care. In addition, there is little evidence to suggest that any of the final 48 apps included in our landscape analysis have been rigorously evaluated for feasibility, clinical trials, or user deployments (eg, none of the apps in our search appeared in any papers in our scoping review). This suggests that to date, the perspectives of HHAs in both the design and evaluation of technology have been lacking.

Rather, our landscape analysis suggests that many of the existing apps have been primarily developed with home care agencies in mind, providing functionality that primarily serves the administrative needs of the agency (eg, electronic visit verification) rather than providing on-the-job support for HHAs. Compounding these concerns, the COVID-19 pandemic has accelerated and amplified the use of technology in HHAs’ work. For example, home care agencies have been rapidly transitioning to using digital tools for remote training, scheduling, and monitoring HHAs’ work, but it remains unclear whether, or to what extent, these technology tools meet HHAs’ needs or impact patient care [27]. However, some of the apps in our study provide functions that support HHAs, including the ability to obtain training, communicate with their agencies or patients, and search for patient information. Additional efforts are needed to understand how HHAs perceive these functions, including how they are used in real time and what their impact is on HHA employment attitudes (eg, job satisfaction) and self-efficacy in providing care.

Notably, several recent reviews of mHealth apps relate to and build on some of our main findings. A scoping review by Vaughan et al [28] analyzed existing studies of mHealth apps that support nurses in monitoring their patients’ chronic wounds. They found that although several wound care apps are available for nurses to use, there is a lack of rigorous and standardized evaluations of these apps, few clinical trials, and a paucity of information about which apps nurses actually use in real time [28]. A scoping review by Dauletbaev et al [29] highlighted the increased the use of mHealth telemonitoring for patient care since the COVID-19 pandemic, a trend we also saw in-home health. Although both reviews highlight the importance of technology for the health care workforce and patient care, neither review included HHAs. Finally, a recent systematic review by Widdison et al [30] examined the effectiveness of randomized control trials that used mHealth apps to deliver pelvic floor muscle training exercises to patients with urinary incontinence. The rigor of the included studies signals a gap with the studies in our review and where the current research on HHAs needs to be conducted in the future.

### Limitations

Our study has a few limitations. First, new studies and mobile apps may have been published or released after we collected data for our scoping review and landscape analysis, which may signal an underrepresentation of existing studies and apps. Second, mobile apps available at the time of data analysis may have been discontinued. Finally, our study examined mobile app descriptions on the Google Play or the Apple stores between 2019 and 2021, and our categorization of these apps depends on the accuracy of these descriptions. Future studies should verify publicly facing descriptions of these apps to confirm that they accurately represent the intended use of the products.

### Conclusions

Our findings suggest that despite the integral role of HHAs in patient care and their exposure to and use of technology, few studies of technology-based interventions designed for this workforce exist and those that do lack rigorous evaluations. In addition, although many apps for the workforce are in use, most are designed from the perspective of the home care agency, not the HHA, and serve to monitor HHAs rather than support them in providing care to patients. Taken together, there is an urgent need for research that centers on the needs and perspectives of HHAs and using human-centered methods to engage HHAs in the design of technologies that truly support their essential caregiving work. Such approaches will also likely make HHAs feel more included and valued in the health care system, addressing the challenges identified in prior work. Therefore, more rigorous evaluations of both existing and new technologies, including clinical trials that effectively measure the impact of the technology on both HHAs and patients for whom they care, are warranted.

## Appendix

Multimedia Appendix 1 Search strategy terms for scoping review.

Multimedia Appendix 2 Full results from the scoping review of literature.

Multimedia Appendix 3 Search strategy terms for landscape analysis.

Multimedia Appendix 4 Full results from the landscape analysis of mobile apps.

## Abbreviations

HHA: home health aide
mHealth: mobile health
PRISMA-ScR: Preferred Reporting Items for Systematic Reviews and Meta-Analyses extension for Scoping Reviews
