# Supplementary material for: Understanding the Technological Landscape of Home Health Aides: Scoping Literature Review and a Landscape Analysis of Existing mHealth Apps
Source: J Med Internet Res. 2022 Nov 11;24(11):e39997. doi: 10.2196/39997 (PMC9700235; doi:10.2196/39997)
Supplement: Multimedia Appendix 1 [file jmir_v24i11e39997_app1.docx]

## Appendix 1. Search Strategy Terms for Scoping Review

Ovid MEDLINE(R) ALL 1946 to October 27, 2020

1 Home Health Aides/

2 Home Care Services/

3 House Calls/

4 Home Health Nursing/

5 (home adj2 (aide* or agencies or attendant* or base* or care or community or communities or nurse* or nursing or personnel or program* or programme* or service* or visit* or worker*)).mp.

6 (domestic health or domiciliary care or house call* or personal care aide* or personal care attendant*).mp.

7 or/1-6

8 Mobile Applications/

9 Computers, Handheld/

10 Smartphone/

11 Cell Phone/

12 Text Messaging/

13 (Android or cell phone* or cellular or ehealth or hand held* or handheld* or handset* or Iphone* or mhealth or mobile app* or mobile application* or mobile device* or mobile health or mobile learning or mobile phone* or mobile technologies or mobile technology or mobile telephone* or palm pilot* or palmtop computer* or palm-top computer* or PDA or personal digital assistant* or pocket pc or pocket pcs or portable apps* or portable electronic app* or portable media device* or portable software app* or short message service or smart phone* or smartphone* or tablet* or text messag* or texting*).mp.

14 technology/

15 Educational Technology/

16 (technology or technologies).mp.

17 or/8-16

18 exp Aged/

19 ("65" or "80" or aged or centenarian* or elder or elderly or frail or geriatric* or nonagenarian* or octogenarian* or old or senior* or senium).mp.

20 or/18-19

21 7 and 17 and 20
