# Supplementary material for: Understanding the Technological Landscape of Home Health Aides: Scoping Literature Review and a Landscape Analysis of Existing mHealth Apps
Source: J Med Internet Res. 2022 Nov 11;24(11):e39997. doi: 10.2196/39997 (PMC9700235; doi:10.2196/39997)
Supplement: Multimedia Appendix 2 [file jmir_v24i11e39997_app2.docx]

**Multimedia Appendix 2**

**Table S1.** Full results from the scoping review of literature.

| Author, year, and country^a^ | Study title | Journal | Technology innovation | Intended users | HHA^b^ role | Study objectives and systems goals | Evaluation and assessment of innovation |
| --- | --- | --- | --- | --- | --- | --- | --- |
| Ogawa et al, 2004; Japan | A Java mobile phone-based “home helper” care report creation support system | *Biomedical Sciences Instrumentation* | Java mobile phone–based care report creation support system | Home helpers | Primary | To describe the technology | Simplified system evaluation; no user evaluation/deployment; system experimental trial with 21 home helper participants |
| Scandurra et al, 2006; Sweden | Visualisation and interaction design solutions to address specific demands in shared home care | *Ubiquity: technologies for better health in aging societies (book)* | Design prototype based on participatory design | GP^c^, DN^d^, and HHS^e^ personnel | Peripheral; one of many | To develop and test a prototype system to improve communication and information flow between home health care and primary health care; sample: 3 HHS personnel, 2 DNs, and 2 GPs | Design prototype; no user evaluation/deployment; not clear how data would be entered into the system by stakeholders |
| Paganelli et al, 2011; Italy | An ontology-based system for context-aware and configurable services to support home-based continuous care | *IEEE Transactions on Information Technology in Biomedicine* | Emilia Romagna Mobile Health Assistance Network (ERMHAN) service platform | Patients, family members, home care teams (clinicians, GPs, nurses, etc), and social community members (eg, social workers and volunteers) | Peripheral; one of many | To describe the key concepts of the design of the ontology-based context model adopted in the ERMHAN platform for the home-based care setting | Design prototype; no user evaluation/deployment |
| Page et al, 2012; the United States | Improving care delivery using health information technology in the home care setting: development of the home continuation care dashboard | *Annals of Long-Term Care* | Web dashboard intended to bridge the gap between physicians and home care case managers | Physicians, home care case managers, patients, and caregivers | Peripheral; one of many | To describe and refine the development of the web-portal, Home Continuation Care Dashboard, designed to facilitate communication among case managers, physicians, patients, and their caregivers at home | Dashboard is described as “still in development”; no user evaluation/deployment; URL for dashboard no longer works |
| De Backere et al, 2016; Belgium | The OCareCloudS project: toward organizing care through trusted cloud services | *Informatics for Health and Social Care* | OCarePlatform and cloud-based semantic system to offer information and knowledge-based services for older people and their informal and formal caregivers | Older patients residing at home and informal and formal caregivers | Secondary; 1 of 3 (patient is primary) | To describe the methodologies to develop a cloud-based semantic system for patients and their caregivers | Design prototype; no user evaluation/deployment |
| De Backere et al, 2017; Belgium | The OCarePlatform: a context-aware system to support independent living | *Computer Methods and Programs in Biomedicine* | Sensor-based in-home system | Multiple formal and informal caregivers involved in a patient’s care | Peripheral; one of many | To describe the design and architecture of the OCarePlatform system | Technical evaluation only; no user evaluation or deployment; the OCarePlatform can be used to respond to a trigger in <5 seconds |
| Danilovich et al, 2017; the United States | Design and development of a mobile exercise application for home care aides and older adult Medicaid home and community-based clients | *Home Health Care Services Quarterly* | Mobile exercise app. Content of the program itself seems static. Minimal data entry about patient (pain and mood) | Home HCA^f^ and patients | Secondary; 1 of 2 (patient is primary) | To describe the HCA, older adult HCBS^g^ clients, and physical therapists in the design, development, and pilot-testing of a mobile exercise app to (1) determine preferred exercise content, (2) identify desired mobile app features, and (3) ascertain exercise training needs | A 2-week pilot study (N=5 dyads); presents user evaluation/deployment from HHAs’ perspective |
| Bourikas et al, 2017; the United Kingdom | Elderly support to inspired ageing (ESTIA) | *International Conference Series on Energy and Cities* | Elderly Support to Inspired Ageing platform that enables medical and background information to be combined into a single server | Family, volunteers, older people, home care aides, hospitals | Primary | To describe the technology | No user evaluation/deployment, very simplified system evaluation |
| Danilovich et al, 2017; the United States | Translating Strong for Life into the Community Care Program: Lessons Learned | *Journal of Applied Gerontology* | SFL^h^: Resistance Exercise Intervention: 35-minute DVD on warm-up and upper and lower extremity exercises for homebound older adult clients | Home HCA and patients | Secondary; 1 of 2 (patient is primary) | To test the feasibility and effectiveness of training HCAs to implement SFL for homebound older adult clients enrolled in the Community Care Program, funded by Medicaid | Mixed methods randomized controlled trial with HCA-patient dyads in the control or intervention groups. SFL training evaluation via qualitative assessments and field observations of HCAs and HCAs’ interactions with their clients through research assistants; program evaluation by HCAs and clients after 1 week of course completion: assessed program satisfaction, benefits, implementation, and suggestions. HCA job satisfaction via quantitative and qualitative assessments |

^a^Studies are listed in chronological order based on the year published.

^b^HHA: Home health aide.

^c^GP: general practitioner.

^d^DN: district nurse.

^e^HHS: home help service.

^f^HCA: health care aides.

^g^HCBS: home and community based services.

^h^SFL: strong for life.
