# Supplementary material for: Understanding the Technological Landscape of Home Health Aides: Scoping Literature Review and a Landscape Analysis of Existing mHealth Apps
Source: J Med Internet Res. 2022 Nov 11;24(11):e39997. doi: 10.2196/39997 (PMC9700235; doi:10.2196/39997)
Supplement: Multimedia Appendix 3 [file jmir_v24i11e39997_app3.docx]

## Appendix 3. Search Strategy Terms for Landscape Analysis

home health care

home care worker

home care attendant

home health aide

home health worker

certified nursing assistant

state certified nursing aide

EVV

track certified nursing assistant's patients

track home care worker's patients

track home health aide's patients

track home health worker's patients

track nurse's patients

track state certified nursing aide's patients
