# Supplementary material for: Understanding the Technological Landscape of Home Health Aides: Scoping Literature Review and a Landscape Analysis of Existing mHealth Apps
Source: J Med Internet Res. 2022 Nov 11;24(11):e39997. doi: 10.2196/39997 (PMC9700235; doi:10.2196/39997)
Supplement: Multimedia Appendix 4 [file jmir_v24i11e39997_app4.docx]

**Multimedia Appendix 4**

**Table S1.** Full results from the landscape analysis of mobile apps.

| Name of the mobile app^a^ | Year | Type | Country | Primary user(s) | Number of downloads | User rating | Total reviews | Developer | Objective |
| --- | --- | --- | --- | --- | --- | --- | --- | --- | --- |
| Domiciliary Care Toolkit | 2014 | Android | Ireland | Home care providers (including HCWs^b^) | >1000 | 4.1 | 8 | Government and software company | Supporting |
| HHAeXchange | 2014 | Android | The United States | HCW | >100,000 | 3.6 | 6086 | Software company | Both |
| Verify Centre Home Health | 2015 | Android | The United States | HCW | >1000 | 3.9 | 24 | Software company | Both |
| Alora Plus | 2016 | Android | The United States | HCW | >10,000 | 3.2 | 77 | Software company | Both |
| Connected Home Care | 2016 | Android | The United States | HCW | >500 | 3.9 | 13 | Agency | Both |
| Electronic Visit Verification | 2016 | Android | Wales | HCW | >1000 | 5 | 5 | Software company | Monitoring |
| FreedomCare Plus | 2016 | Android | The United States | HCW | >10,000 | 3.7 | 1061 | Agency | Monitoring |
| MedFlyt | 2016 | Android | The United States | Home care providers (including HCWs) | >50,000 | 4.7 | 2466 | Software company | Both |
| PointClickCare Care at Home | 2016 | Android | The United States and Canada | HCW | >10,000 | 3.8 | 63 | Software company | Monitoring |
| CareConnect | 2017 | Android | The United States | HCW | >10,000 | 4.1 | 1226 | Software company | Supporting |
| Axxess HomeCare | 2017 | Android | The United States | HCW | >10,000 | 4 | 218 | Software company | Both |
| Caretap EVV | 2017 | Android | The United States | Home care providers (including HCWs) | >1000 | 3.2 | 19 | Software company | Monitoring |
| DCI Mobile EVV | 2017 | Android | The United States | HCW | >10,000 | 2.8 | 822 | Software company | Both |
| eRSP Mobile Connect | 2017 | Android | The United States | Home care providers (including HCWs) | >50,000 | 4.3 | 454 | Software company | Both |
| FormDox EVV for Aides | 2017 | Android | The United States | HCW | >500 | 4.4 | 34 | Software company | Both |
| Ally Home Care | 2018 | Android | The United States | HCW | >1000 | 3.2 | 14 | Software company | Monitoring |
| August Systems Mobile for Caregivers | 2018 | Android | The United States | HCW | >5000 | 3.4 | 36 | Software company | Both |
| AuthentiCare 2.0 | 2018 | Android | The United States | HCW | >50,000 | 2.6 | 1477 | Software company | Monitoring |
| ClearCareGo Caregiver | 2018 | Android | The United States | HCW | >100,000 | 3.6 | 18,054 | Software company | Monitoring |
| CliniqOS | 2018 | Android | The United States | Home care providers (including HCWs) | >100 | —^c^ | — | Software company | Both |
| CrescendoConnect | 2018 | Android | The United States | Home care providers (not specific to HCWs) | >5000 | 3.1 | 54 | Software company | Both |
| Domiciliary Care Worker Gweithiwr Gofal Cartref | 2018 | Android | Wales | HCW | >500 | — | — | Government and agency | Supporting |
| Helpers Home Care | 2018 | Android | Ethiopia | Home care providers (including HCWs) | >100 | 5 | 5 | Agency | Both |
| My EVV | 2018 | Android | The United States | HCW | >10,000 | 3.9 | 407 | Agency | Monitoring |
| MyEzcare—EVV | 2018 | Android | The United States | HCW | >1000 | 4.1 | 88 | Software company | Monitoring |
| Mobile Caregiver+ | 2018 | Android | The United States | HCW | >10,000 | 3 | 1959 | Software company | Monitoring |
| Honor Care Pro | 2018 | Android | The United States | HCW | >5000 | 4.4 | 356 | Agency | Both |
| UCP Caregiver Staffing | 2018 | iOS | — | HCW | — | — | — | — | Monitoring |
| BarbaraKares | 2019 | Android | The United States | HCW | >50 | — | — | Agency | Monitoring |
| CareTime | 2019 | Android | The United States | HCW | >5000 | 2.8 | 48 | Software company | Monitoring |
| Cashe EVV | 2019 | Android | The United States | HCW | >1000 | 2 | 30 | Software company | Both |
| KorEvv | 2019 | Android | The United States | HCW | >100 | 5 | 1 | Software company | Monitoring |
| MatrixCare for Home Care | 2019 | Android | The United States | HCW | >10,000 | 3.5 | 121 | Software company | Both |
| myHRresults—At Work | 2019 | Android | — | HCW | >5 | N/A^d^ | 0 | — | Monitoring |
| SwyftOps—Caregiver App | 2019 | Android | The United States | HCW | >5000 | 3.4 | 33 | Software company | Monitoring |
| Vertex EVV | 2019 | Android | The United States | HCW | >100 | 5 | 1 | Software company | Monitoring |
| HomecareGPS Mobile | 2019 | iOS | The United States | HCW | — | 5 | 1 | Software company | Monitoring |
| ServTracker Mobile Home Care | 2019 | iOS | The United States | HCW | — | 3 | 2 | Software company | Monitoring |
| Moravia Shifts | 2020 | Android | The United States | HCW | >10 | NA | 0 | Agency | Monitoring |
| Netsmart Homecare Mobile Phone | 2020 | Android | The United States | HCW | >1000 | 3.4 | 5 | Software company | Both |
| BAYADA Home | 2021 | Android | The United States | HCW | >5000 | 3.6 | 15 | Agency | Monitoring |
| Careswitch | 2021 | Android | The United States | HCW | >100 | — | — | Software company | Both |
| Visit Wizard Mobile | — | Android | The United States | HCW | >1000 | 3.25 | 4 | Software company | Both |
| Best Care | — | Android | The United States | HCW | >1000 | 3.6 | 21 | Software company | Monitoring |
| Caregiver App | — | Android | The United States | HCW | >10,000 | 3.4 | 69 | Agency | Monitoring |
| Caregiver Cloud Training | — | Android | The United States | Home care providers (including HCWs) | >1000 | — | — | Software company | Supporting |
| Time4Care | — | Android | The United States | HCW | >100,000 | 2.8 | 4 | Software company | Monitoring |
| ViolaCare | — | Android | — | HCW | >10 | N/A | 0 | — | Monitoring |

^a^Apps are listed in chronological order based on the year created or last updated.

^b^HCW: home care worker

^c^Missing information.

^d^N/A: not applicable.
